# Lactylation and Central Nervous System Diseases

**DOI:** 10.3390/brainsci15030294

**Published:** 2025-03-11

**Authors:** Ye Chen, Dongqiong Xiao, Xihong Li

**Affiliations:** 1 Department of Emergency Medicine, West China Second University Hospital, Sichuan University, Chengdu 610041, China; chenye@scu.edu.cn (Y.C.); xiaodq5221@scu.edu.cn (D.X.); 2 Key Laboratory of Birth Defects and Related Diseases of Women and Children, Sichuan University, Ministry of Education, Chengdu 610041, China

**Keywords:** lactate, lactylation, central nervous system diseases

## Abstract

As the final product of glycolysis, lactate serves as an energy substrate, metabolite, and signaling molecule in various diseases and mediates lactylation, an epigenetic modification that occurs under both physiological and pathological conditions. Lactylation is a crucial mechanism by which lactate exerts its functions, participating in vital biological activities such as glycolysis-related cellular functions, macrophage polarization, and nervous system regulation. Lactylation links metabolic regulation to central nervous system (CNS) diseases, such as traumatic brain injury, Alzheimer’s disease, acute ischemic stroke, and schizophrenia, revealing the diverse functions of lactylation in the CNS. In the future, further exploration of lactylation-associated enzymes and proteins is needed to develop specific lactylation inhibitors or activators, which could provide new tools and strategies for the treatment of CNS diseases.

## 1. Introduction

In 1780, a Swedish pharmacist assistant, Carl Wilhelm Scheele, discovered lactate in yogurt [1]. In 1808, the Swedish chemist, Jons Jacob Berzelius, discovered that lactate is produced in the muscles of animals during exercise and hypothesized that this acid is found in muscles and in yogurt post the discovery by Carl Wilhelm Scheele [2]. Lactate was long believed to serve no biological purpose and was considered a waste product of glycolysis. However, recent studies have demonstrated that lactate plays a role in signal transduction, regulation, and energy metabolism [3].

Lactate is a monocarboxylic acid with the molecular formula C_3_H_6_O_3_. It contains hydroxyl groups and belongs to the alpha-hydroxy acid family. Based on optical rotation, it is divided into two configurations, namely L- and D-type. L-lactate is distributed in various cells, tissues, organs, and the whole body, whereas D-lactate is uncommon in mammalian metabolism. Lactate is one of the key molecules in the metabolism of glycolysis. Hexokinase, phosphofructokinase-1, and pyruvate kinase (PK/PYK) catalyze the conversion of glucose, which enters the cells through glucose transporters, into pyruvate. Pyruvate dehydrogenase catalyzes the transformation of pyruvate into acetyl-CoA in aerobic environments. After entering the tricarboxylic acid (TCA) cycle, acetyl-CoA undergoes oxidative phosphorylation (OXPHOS) to generate ATP. The interconversion between lactate and pyruvate is predominantly mediated by lactate dehydrogenase (LDH). Under anaerobic conditions, LDH catalyzes the reduction of pyruvate to lactate. Conversely, under aerobic conditions, LDH facilitates the re-oxidation of lactate to pyruvate [4]. Therefore, lactate is a key metabolite between glycolysis and OXPHOS [5].

This paper systematically elucidates how lactylation modification drives the progression of CNS diseases through a “metabolite (lactate)-epigenetic regulation (histone modifications)-neuroimmune microenvironment remodeling (e.g., microglial polarization)” cascade mechanism. Building on previous studies, we propose the “double-edged sword effect” of Kla: Depending on the disease context or stage of neuroinflammation, lactylation exerts divergent roles. For instance, short-term activation of glycolysis enhances microglial functionality (e.g., phagocytic clearance of pathological proteins), whereas prolonged lactate accumulation and elevated Kla levels disrupt microglial metabolic homeostasis, leading to dysfunction and exacerbated neuroinflammation (e.g., sustained amyloid-β deposition in Alzheimer’s disease). Investigating the relationship between lactate and lactylation not only helps to elucidate the pathogenesis of CNS diseases but also provides an important theoretical foundation for the development of novel therapeutic strategies.

## 2. Role of Lactate in the Central Nervous System

**Figure 1:**
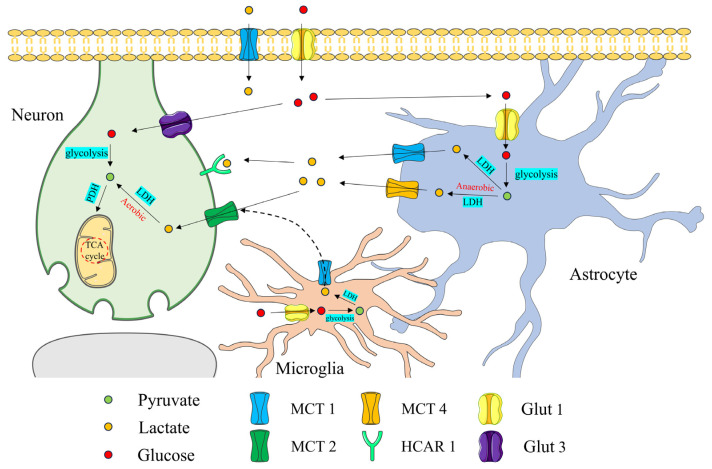
Lactate functions within the CNS as follows: During periods of high neuronal energy demand, such as increased synaptic activity, astrocytes rapidly supply lactate to neurons through the ANLS mechanism, thereby supporting neuronal function. Glut1 mediates the transfer of glucose into the cytoplasm, where it undergoes glycolysis in astrocytes to produce pyruvate. LDH catalyzes the conversion of pyruvate to lactate, which is then transported to the astrocyte–neuron gap via MCT1 and MCT4. Subsequently, it is taken up by neurons through MCT2. Within neurons, lactate can be oxidized back to pyruvate by LDH to fuel the tricarboxylic acid (TCA) cycle, thereby providing energy for neuronal function. Glut3 mediates the transport of glucose into neurons, providing energy for them. Microglia can also produce lactate through glycolysis, but whether this lactate can directly supply energy to neurons remains to be further investigated.

Lactate has several functions. It acts as (1) the main source of energy, (2) the main precursor of gluconeogenesis, and (3) a signaling molecule [6]. As the end result of glycolysis metabolism, lactate serves as both an intercellular signaling molecule and an energy substrate for neuronal activity in the brain. In the brain, astrocytes can secrete lactate to neighboring neurons, which utilize lactate and convert it into pyruvate to produce energy (Figure 1) [7]. Thus, lactate can serve as an energy substrate in the brain. The change in its concentration correlates with neuropsychiatric diseases. Lactate affects neuron excitability, regulation of pH in the brain, body fluid balance, neurovascular coupling, and long-term memory formation [8]. Therefore, lactate may be a potential target for the treatment of CNS diseases.

### 2.1. Lactate Promotes Basic Nerve Function and Development

During brain nerve development, axon elongation and dendritic development are key processes in the establishment of neural networks. In vitro studies have revealed that exogenous lactate administration reverses the growth and development of neuronal axons and dendrites when lactate production and release in astrocytes are interfered with. This suggests that lactate plays a significant role as an energy substrate in nerve development [9]. Astrocyte-derived lactate is crucial for high-energy activity in Drosophila neurons [10]. The brain regulates its own lactate supply through glycolysis and glycogen decomposition in astrocytes. Studies have shown that the cerebral cortex can maintain normal synaptic activity with only pyruvate and lactate as metabolic substrates, and lactate can independently support hippocampal activity and the survival of cultured neurons in vitro [11,12].

Development of the myelin sheath is necessary to maintain the long-term stability of axons. Oligodendrocytes are the main glial cells that affect the formation of the myelin sheath. Upon increasing the rate of glycolysis in oligodendrocytes, the metabolite lactate provides fuel to the axons. Interfering with oligodendrocyte formation affects neuronal survival and axonal function, indicating that oligodendrocyte-derived lactate provides support for axonal energy supply [13]. Many neurodegenerative diseases, including multiple sclerosis and amyotrophic lateral sclerosis, begin with rapid axon loss [14]. Understanding the role of lactate as an energy source provides a theoretical basis for early clinical intervention and treatment in neurodegenerative diseases [15]. In the brain, lactate functions as a neurotransmitter and a vital energy source. Lactate and its regulating variables are expected to play significant roles in the prevention and management of diseases affecting the neurological system [16].

### 2.2. Lactate Regulates Brain pH

Lactate plays multiple important roles in the physiological activities of the brain [17]. The glycolytic breakdown of glucose in astrocytes is assumed to be the primary source of L-lactate in the brain, whereby L-lactate predominates and D-lactate is nonexistent [18,19]. The astrocyte–neuron lactate shuttle (ANLS) hypothesis states that astrocytes spontaneously convert glucose or glycogen into lactate in response to signals from neuronal activity (Figure 1). The monocarboxylate transporter (MCT) subsequently uses this lactate to fuel neurons. This hypothesis emphasizes the key function of lactate in brain metabolism [20].

Because enzyme activity and protein folding are highly sensitive to pH variations, brain pH is one of the most significant factors that can affect the initiation and duration of pathophysiological processes in patients with CNS diseases [21]. Many CNS proteins, such as those involved in synaptic transmission, ion channels, and neurotransmitter receptors, are extremely sensitive; therefore, even small changes in brain pH can have a significant impact on various signaling pathways, cell membrane excitability, action potential generation, and synaptic activity. This is because lactate levels are one of the factors that influence brain pH, and changes in pH affect neuron activity [22]. Changes in energy metabolism and mitochondrial malfunction may be the cause of lactate-mediated pH shifts in the brains of patients with neuropsychiatric diseases, such as autism [23,24]. Astrocytes are in charge of restoring the pH balance. By producing bicarbonate, which is reliant on neuronal activity, they can restore the local extracellular pH and prevent acidification of the brain environment [25].

### 2.3. Lactate Participates as a Signaling Molecule in the Physiological Regulation of the CNS

MCTs belong to the solute carrier family 16, which is composed of 14 members, named MCT1–14 [26]. However, only MCT1–4 have been extensively studied and identified as being involved in lactate transport [27]. MCT isoforms have different affinities for lactate, and their distribution is cell-specific. Astrocytes mainly express MCT1 and MCT4 with low affinity, and neurons express MCT2 [28,29]. MCT3 is mainly expressed on choroid plexus epithelial cells and retinal epithelial cells [30]. The transmembrane transport of lactate is mostly mediated by MCT1, which has a high affinity for lactate. MCT1 protein levels fluctuate in tandem with variations in lactate levels [31]. MCT4 is primarily in charge of the transmembrane export of lactate and has a modest affinity for it [32]. However, in cells with high glycolysis activity, MCT1 can achieve the transmembrane export of lactate because the transfer or export of lactate depends on the lactate concentration gradient [33]. In the brain, astrocyte lactate can be broken down by neurons, and astrocytes absorb the glutamate released by neurons [34]. Therefore, glycolysis is activated again in the astrocytes; lactate moves to the extracellular space through MCT4 and is taken up into the cell by MCT2 on the neuron membrane to provide an energy substrate (Figure 1) [35].

Certain receptors in the brain, primarily the G protein-coupled receptor (GPCR), commonly referred to as hydroxycarboxylic acid receptor 1, are necessary for the action of lactate [36]. GPCR is the largest known type of seven-fold transmembrane receptor that binds various ligands to activate signal cascades. Lactate is one of the endogenous ligands of GPCR and is involved in regulating various immune processes, such as cytokine production, immune tolerance, and disease occurrence, through GPCR 81 (GPR81) and GPCR 132 (GPR132) [37]. In the brain, GPR81 is expressed by various cell types, including neurons, astrocytes, and microvessel cells. Lactate binds GPR81 to control intracellular second messengers [38]. By controlling the movement of lactate between the inner cellular compartment and the plasma membrane, the lactate receptor GPR81 prevents protein kinase A- and cAMP-mediated signaling [39]. In addition, lactate can induce vascular regeneration and epileptogenesis in the brain through GPR81 and affect a range of central activities, such as neural activity and neurogenesis.

Because lactate can cross the blood–brain barrier, lactate generated outside the CNS from food and exercise impacts the brain and how brain cells operate [40]. In the mouse hippocampus, lactate content increased dramatically after intraperitoneal lactate injection, suggesting that peripheral lactate injection crossed the blood–brain barrier and affected hippocampal tissue [41]. The primary functioning cells in the brain that produce lactate are called astrocytes. Glucose transporter 1 (Glut1) carries glucose from the interstitial space into astrocytes. When neighboring neurons require increased energy supply, astrocytes rapidly activate their intrinsic glucose reserves and metabolic pathways, where glycolysis produces pyruvate, which is then oxidized to generate energy [42]. Under anaerobic conditions, pyruvate in astrocytes is reduced to lactate by LDH and subsequently released into the extracellular space via MCT1 and MCT4. Lactate, a signaling molecule in the interstitial region, binds to the lactate receptor on the neuronal cell membrane, known as hydroxycarboxylic acid receptor 1 (HCAR1). Additionally, MCT2 transports lactate into neurons, where, under aerobic conditions, lactate can be oxidized to pyruvate by LDH to fuel the TCA cycle, thereby providing energy for neuronal function [43]. Glut3 transports glucose from the interstitial space to neurons, supplying them with energy [44]. The ANLS hypothesis explains the coupling between neuronal synaptic activity and astrocytic glycolytic metabolism. Although microglia can also generate lactate through glycolysis, the primary source of lactate for neuronal function is believed to be astrocytes [45]. Whether lactate produced by microglia directly supports neuronal energy demands remains to be further investigated. Lactate plays a critical role in CNS diseases by modulating the metabolic and inflammatory states of the microglia–neuron–astrocyte network (Figure 1).

## 3. Lactylation

### 3.1. Histone Lactylation

Neural plasticity depends on both transient and permanent alterations in gene expression. By forming modification or cleaving groups through proteolysis, histones attach to specific amino acid residues through covalent bonds, altering protein characteristics and controlling gene expression. These are called post-translational modifications (PTMs) [46]. Histones are an important part of chromatin and, together with DNA, constitute the nucleosome structure. The core histone is composed of two H3–H4 tetramers and two H2A–H2B dimers (co-octamers) combined to form a nucleosome core, and DNA is wrapped around the nucleosome core [47]. Important for the assembly of nucleosomes, the C-terminus of core histones stabilizes the histone structure and encourages connections between histones and DNA. The PTM sites of histones are located in the N-terminal region, which also has a flexible charged area at the end. PTMs control how well histones attach to DNA and impact chromosome shape, nucleosome stability, and chromatin structure [48].

In 2019, a new PTM, histone lysine lactylation (Kla), was proposed [49]. Researchers have hypothesized the presence of a “lactate clock” in cells. When a certain amount of exogenous or endogenous lactic acid is accumulated, lactic acid initiates active enzymatic PTM with lactyl-CoA as substrate. After the “writer” adds a lactate group to the histone lysine residue, the lactate group is covalently bound with the histone to change the tightness of the histone and DNA, indirectly controlling the expression or silencing of certain genes. When the downstream gene is activated, the “eraser” removes the lactate group from the lysine residue, which is essential for maintaining protein function and cell metabolism [50]. Writers and erasers related to Kla are acetyltransferase P300 and histone deacetylase (HDAC), respectively [51].

The epigenetic functions of the write, read, and erase enzymes can control the histone Kla process [52,53]. The level of histone Kla is proportional to the intracellular lactate content. Reduced production of lactate, including the inhibition and knockout of enzymes related to glycolysis, such as the M2 isoform of PK (PKM2) and LDHA, can significantly inhibit intracellular histone Kla [54]. Inhibition of the aerobic respiratory pathway increases intracellular lactate and histone Kla levels [55]. Elevated extracellular lactate concentrations increase intracellular lactate and histone Kla levels [56]. It is evident that lactate is a crucial histone Kla regulator that directly impacts its levels.

**Figure 2:**
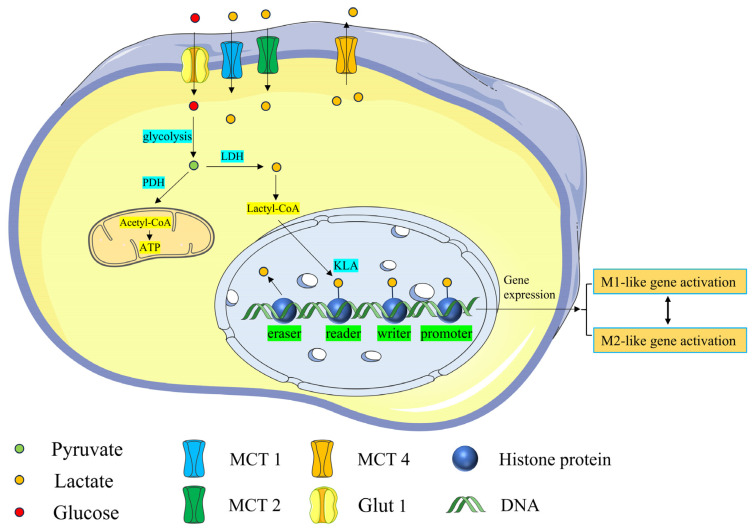
The routes of intracellular lactylation and lactate generation are as follows: Extracellular lactate enters cells through MCT1 and MCT2, while MCT4 transports intracellular lactate outside the cells. In the cytoplasm, lactate is converted to lactyl-CoA. Through a specific enzymatic reaction, lactyl-CoA mediates histone Kla by transferring lactate groups to lysine residues in histones. This process is regulated by enzymes that act as writers, readers, and erasers. Histone Kla regulates gene transcription in the promoter region, influencing both the M1 and M2 phenotypes of macrophages.

Brain cells contain histone Kla, and astrocytes, microglia, gamma-aminobutyric acid neurons, and glucose–aminobutyric acid neurons exhibit the immunological response of Kla [56]. The excitatory stimulation of neurons can increase lactate levels in the brain, and the degree of Kla is dependent on lactate concentration [57]. These histone modifications are crucial for neuron formation, synaptic plasticity, and behavioral memory in the CNS. They can also act as regulators of gene expression, enabling cells to adjust to environmental changes [58]. Kla is an important mode for lactate to function and is involved in key processes, such as macrophage polarization, vascular remodeling, mitochondrial function, and nervous system regulation, opening up new directions for future research [59] (Figure 2).

### 3.2. Nonhistone Lactylation

Kla is present in histones and nonhistones, but nonhistone lysine residues are more difficult to undergo lactyl substitution [60]. Most lactylated proteins in Botrytis cinerea are found in the cytoplasm, mitochondria, and nucleus and are involved in many cellular functions. Kla is crucial for ribosome assembly and controlling protein translation [61]. This parasite contains a range of lactate proteins that play a significant role in RNA export, translation, degradation, trans-splicing, and cap binding [62]. Most key enzymes involved in glycolysis can undergo lactylation, including phosphoglycerate kinase and PYK [63]. Kla is widely present in various proteins and is a common PTM. Lactylation of non-histones is less studied, but it may play an important role in cellular regulation.

## 4. Kla and CNS Diseases

The regulation of gene-specific expression depends on the cross-regulation of diverse epigenetic modifications and molecules that bind to them. This process establishes the chromatin state of distinct genomic regions. Dysregulation of metabolic activity and epigenetic changes are frequently linked to various diseases [64]. Because aberrant epigenetic mechanisms can result in various diseases and may include numerous new therapeutic targets, epigenetic regulation governs the establishment and modification of cellular functional states in various physiological and pathological processes. This makes it crucial for research on maintaining health and disease development [65]. Histone PTMs are implicated in several facets of neuronal function and development, and epigenetic mechanisms play major roles in maintaining a healthy brain [66].

Lactate ameliorates memory loss, increases cerebral blood flow, improves brain energy metabolism, lessens neurological dysfunction, and encourages nerve regeneration, among other benefits [42,67]. Kla draws attention to the novel function of lactate in controlling transcription, cellular activity, and disease onset. Since Kla discovery, studies have evaluated the role of lactate in the CNS. Neuronal death and atrophy in certain brain regions as well as the disintegration of neural networks due to progressive neuronal death are characteristics of CNS diseases [68]. The synthesis of cytokines and chemokines by glial (microglia and astrocytes), endothelial, and peripherally derived immune cells of the the CNS mediates neuroinflammation (inflammatory response in the CNS). Acute neuroinflammation protects the CNS from poisons, traumas, and infections. Microglia are innate immune cells that are crucial for damage responses, neurodegenerative diseases, and preserving the homeostasis of the CNS [69]. Microglia dysfunction destroys the CNS, leading to disease development, attributable to mitochondrial dysfunction and lactate accumulation [70]. Increased lactate levels facilitate the shift in the polarization balance of microglia through Kla and act as an “accelerator” of the endogenous “lactate timer” of microglia [71]. Lactate may increase histone Kla, which could contribute to the pathophysiology of CNS diseases (Table 1) [72].

### 4.1. Traumatic Brain Injury (TBI)

TBI—a temporary or permanent brain dysfunction brought on by external factors—is one of the primary causes of disability and death in patients with trauma [82]. TBI can lead to cognitive dysfunction and impaired exercise ability. During this process, factors such as mechanical stress, ischemia, and hypoxia act together to cause the destruction of the structural integrity of brain tissue and damage the microstructure, which is represented by neuron cell bodies, axons, dendrites, and myelin sheath, as well as secondary injuries, such as cerebral hemorrhage and cerebral edema. In the early stages of TBI, potential sharp increases in energy requirements and insufficient cerebral blood flow lead to dysfunction. Long-term activation of immune cells amplifies the inflammatory cascade, exacerbating neuronal damage or death, which may lead to severe brain dysfunction. During TBI, particularly during the acute period, trauma-mediated ischemia and hypoxia in brain tissue lead to increased lactate production, to manage the urgent need for energy metabolism in the brain. Its concentration increases significantly after TBI, and it participates in the inflammatory response in the brain as an important immune regulator rather than as a metabolic waste [83]. Importantly, histone Kla serves as a new form of lactate-derived histone PTM that allows lactate to participate in the regulation of complex pathophysiological processes in the CNS [84]. Lactate acts as a signaling molecule in inflammatory lesions in brain tissue. Invasive immune cells can respond to lactate production at inflammatory sites, thereby exerting an anti-inflammatory effect [85]. Thus, studies on the mechanism of action of histone Kla and its immunological modulation during TBI could yield information for early intervention and improve prognosis in patients with TBI.

#### 4.1.1. Kla Regulates the Macrophage Phenotype

TBI triggers the body’s defense response at the site of injury through neuroinflammation. Neuroinflammation is an important defense measure for the body to manage external or internal stimuli; however, long-term neuroinflammation is an important manifestation of brain damage and instability. As a result, one of the key factors affecting the severity of TBI is the immune system’s imbalance following injury. According to recent findings, histone Kla modifications are crucial for controlling immune cell functions, as seen by the increase in inflammation-mediated lactate production as well as the control of macrophage phenotype and microglial metabolic transformation [86].

Microglia can be classified into M1 (pro-inflammatory) and M2 (anti-inflammatory) phenotypes based on their activation status and functions, and these two phenotypes play different roles in the CNS [60]. M1 microglia mainly participate in inflammatory responses and pathogen clearance, while M2 microglia focus on anti-inflammation, tissue repair, and neuroprotection [87,88]. In the early stage of cerebral hypoxia, glycolysis produces lactate, microglia tend to polarize to the M1 phenotype, and M1 microglia release pro-inflammatory cytokines that damage surrounding neurons and glial cells [89]. Lactate inhibits immune cells, such as dendritic, T, and natural killer cells [90]. Kla plays an important regulatory role in TBI and neuroinflammation [91]. Kla may enhance the anti-inflammatory phenotype of macrophages in the brain and promote glycolysis and activation of microglia through a positive feedback cycle, which is conducive to the regression of the inflammatory environment or rapid clearance of disabled cells and promotes restoration of the stable state of the microenvironment after brain injury. The resolution of inflammation and homeostasis equilibrium in the internal environment of the brain depends on the microglia’s ability to control the change in the macrophage phenotype from a proinflammatory to an anti-inflammatory state.

Microglia, resident macrophages in the CNS, play an important role in neuroinflammation and TBI. When the CNS is damaged, microglia quickly activate and trigger a series of adaptive changes, including metabolic reprogramming [92]. Microglia can meet stress-induced energy demands and enhance immune function by upregulating the expression of glycolysis genes [93]. Although short-term enhancement of glycolysis is beneficial for microglial function, long-term lactate production affects the metabolic balance of microglia, leading to microglial dysfunction and neuroinflammation. Therefore, detailed understanding of the Kla pathways and mechanisms is expected to expand prospects for the diagnosis and treatment of TBI.

#### 4.1.2. GPR81 and Brain-Derived Neurotrophic Factor (BDNF)

In various CNS regions, lactate functions as a signaling chemical with its receptor GPR81, potentially enhancing synaptic function, controlling cerebral blood flow, and regulating energy demand [94,95]. Disorders of brain energy metabolism, lack of neurotrophic support, mitochondrial dysfunction, and harm to synaptic plasticity are all part of the pathophysiology of TBI [96]. Lactate is a crucial energy substrate in the CNS and was recently shown to promote synaptic activation. Exogenous lactate supplementation demonstrates therapeutic and protective effects in many models of neuronal disease, including stroke and TBI, and lactate is believed to be crucial for the metabolic support of axons [97,98]. Patients with TBI have higher levels of GPR81 mRNA expression in their ipsilateral hippocampal and cortical regions [99]. By activating the GPR81 receptor, lactate acts as a volume transmitter that links brain energy metabolism, neuronal activity, and energy substrates. Lactate activates GPR81 in the injured brain, reducing poor synaptic function and promoting neuronal protection [73]. Exogenous lactate intervention increased GPR81 expression and aided in the recovery of neurological functioning in TBI rats with cognitive impairment [74]. Lactate exerts a neuroprotective effect on TBI by activating GPR81, which explains how lactate pretreatment affects TBI and offers a therapeutic option in patients with TBI.

A member of the neurotrophic factor family, BDNF is crucial for synaptic plasticity, neuronal development, and the survival of existing neurons. BDNF plays a role in neuroprotection, neuron regeneration, and functional recovery following TBI. Lactate increases intracellular cAMP levels and activates adenylate cyclase when it interacts with GPR81. Protein kinase A is further activated by cAMP, which results in the transcription and translation of BDNF [100]. The ability of lactate to increase BDNF may be connected to the GPR81 signaling pathway. The presence of histone Kla modifications links the control of gene expression to the increase in lactate concentration following TBI. Further elucidating the regulatory role of lactate accumulation in the pathogenic course of TBI will require a thorough investigation of the role of histone Kla.

### 4.2. Alzheimer’s Disease (AD)

The complex process of cell aging comprises many variables that eventually cause the cell cycle to irreversibly stagnate due to a decrease in cell growth and proliferation. AD is an age-related neurodegenerative illness that causes cognitive decline over time [101]. The accumulation of neurofibrillar tangles of tau protein and plaques of amyloid beta (Aβ) protein are two common clinical indicators of AD [102].

The role of neuroinflammation in AD has been gaining increased attention. Brain microglia are immune cells that help with myelin sheath production, synaptic pruning, and the removal of misfolded proteins and cell debris. These are crucial for the development of the brain, preservation of its physiological processes, and onset and progression of different brain diseases. Numerous facets of neuroinflammation, including pathogen defense, damage responses, and CNS tissue maintenance, are influenced by microglia [103]. In addition to releasing inflammatory factors and bioactive chemicals that can harm neurons and accelerate the course of AD, activated microglia exhibit aberrant shape and proliferation [104]. Microglia are of two types: proinflammatory and anti-inflammatory or repair. Repair microglia release receptors and cytokines that reduce inflammation and generate equilibrium [105]. One of the characteristics of AD is the proinflammatory activation of microglia, which involves the switch from OXPHOS to glycolysis [106]. One outcome of this metabolic change is that microglia may generate a lot of glycolysis metabolites and ATP rapidly, which may help microglia’s immunological function. Nevertheless, research has demonstrated that ongoing glycolysis impairs microglial activity, as seen by decreased antibody phagocytosis and migratory activities, suggesting that microglial function is compromised once glycolysis metabolism is triggered [107]. The microglia-mediated neuroinflammatory response puts neurons at even greater risk, which accelerates AD development. Additionally, lactate-derived Kla can exacerbate AD progression [108]. Thus, addressing issues related to lactate metabolism could be a novel approach to AD treatment.

#### 4.2.1. Glycolysis/H4K12 La/PKM2

Abnormal brain glucose metabolism in neurodegenerative diseases can result in issues with energy supply. For instance, in the early stages, patients with AD experience issues with glucose metabolism. The hallmarks of AD include reduced tricarboxylic acid cycle activity, decreased glucose absorption, mitochondrial malfunction, and disruption of energy support, which are provided by astrocytes and oligodendrocytes to neurons [109]. At the same time, neuroinflammation can promote the competition of microglia for glucose, further intensifying the hypometabolism of glucose in the neurons. In AD, synaptic dysfunction and neuronal death can result from cerebral glucose hypometabolism, which exacerbates energy insufficiency and the buildup of neurotoxic proteins. Aβ and tau protein clearance in the brain is impacted by decreased neuronal absorption of glucose and inadequate mitochondrial energy production, whereas the buildup of Aβ and tau causes mitochondrial damage, disrupts energy production, and increases oxidative stress [110]. In AD, this vicious cycle leads to a decline in memory and cognitive abilities and may cause abnormal behavior in patients.

PKM2 is a glycolysis enzyme. A proteome investigation of the postmortem human brain and CSF revealed that AD samples had high levels of PKM2, which was linked to the activation of microglia [111]. Accordingly, research has shown that PKM2 is upregulated in microglia next to Aβ plaques in brain tissues from both patients with AD and an AD mouse model (5XFAD). According to Pan et al., the pathophysiology of AD in microglia is driven by the glycolysis/H4K12 lactylation (H4K12la)/PKM2 positive feedback loop [54]. Elevated histone Kla levels were found in the brain tissues of patients with AD and AD model mice. Among them, H4K12la was significantly elevated in microglia surrounding Aβ amyloid plaques. During the pathological progression of AD, the metabolic profile of microglia undergoes significant changes, shifting from mitochondrial-dependent OXPHOS to a glycolysis-dominant metabolic mode. This metabolic reprogramming enables microglia to rapidly generate ATP to meet the high energy demands during acute inflammatory responses. However, sustained activation of glycolysis impairs microglial phagocytic and chemotactic functions, thereby exacerbating AD progression [112]. The glycolysis activity of the microglia was enhanced by H4K12la, which was abundant in the glycolysis-related gene promoter area of the microglia. The metabolic abnormality of microglia creates a glycolysis/H4K12la/PKM2 positive feedback regulatory loop that intensifies microglia activation and dysfunction in AD. Knocking down PKM2 or interrupting this loop with PKM2 inhibitors can inhibit the proinflammatory activity of microglia, restore their functional stability, reduce neuroinflammatory responses, improve AD pathology, and enhance patients’ learning and memory. In mouse models of AD, PKM2-specific deletion in microglia lowered Aβ burden and enhanced spatial learning and memory, indicating that disrupting the glycolysis/H4K12la/PKM2 positive feedback loop may offer useful therapeutic approaches for AD [54].

In conclusion, Pan et al. discovered that the prefrontal cortex and hippocampus of 5XFAD mice had higher levels of lysine Kla and H4K12la. Glycolysis gene promoters are abundant in H4K12la, which also stimulates the transcription of glycolysis genes. This implies that the glycolysis/H4K12la/PKM2 positive feedback loop mediates long-term neuroinflammation in AD, indicating that blocking the glycolysis–lactate–Kla–glycolysis feedback loop could be a useful treatment approach for AD [113].

#### 4.2.2. H3K18

Aged microglia exhibit a considerable increase in lactate levels, and this buildup raises the level of histone Kla, which accelerates the development of AD pathogenesis and brain aging [114]. In both 5XFAD and naturally aging mice, elevated lactate levels stimulated H3K18 lactylation (H3K18la) in the hippocampus and microglia. When LDH inhibitors were used to treat aged microglia, lactate levels dropped precipitously, which in turn caused histone Kla to significantly decline. Aging microglia and the hippocampal regions of AD and naturally aging animals exhibit markedly elevated levels of H3K18la. Aging and AD phenotypes are promoted by enhanced H3K18la, which directly activates NFκB activation and the senescence-associated secretory phenotype (SASP) [75]. In conclusion, these results imply that the H3K18la/NFκB axis promotes brain aging and the pathological phenotypes of AD by controlling the SASP components IL-6 and IL-8, which in turn control aging-linked inflammation. According to this study, the pathophysiology of AD and brain aging is driven by a positive feedback loop involving the H3K18la/NFκB axis/SASP. These findings offer avenues for developing therapies that target AD pathogenesis and brain aging. By controlling important genes for glycolysis enzymes, such as PKM2, H4K12la contributes to AD pathogenesis, whereas H3K18la does the same by controlling signaling pathways linked to inflammation, such as NFκB. In conclusion, distinct histone lactylation influences AD symptoms and brain aging through several pathways and target genes or pathway regulation in microglia.

#### 4.2.3. Isocitrate Dehydrogenase 3β (IDH3)

Aerobic glucose oxidation involves mitochondria, including the respiratory chain OXPHOS and the TCA cycle. In patients with AD, the TCA cycle is primarily impacted by issues with glucose metabolism. IDH3, a rate-limiting enzyme in the TCA cycle, oxidatively decarboxylates isocitrate to alpha-ketoglutarate and CO_2_ and transforms NAD^+^ into NADH. Protons are taken up by the respiratory chain through this mechanism, which supports OXPHOS and the energy supply [115]. IDH3 comprises a β structural component, a γ conversion subunit, and two α catalytic subunits. It is inert in its monomer state and becomes fully active when heterotetramers are produced in a 2:1:1 ratio. IDH3β plays a key role in energy metabolism and is essential for heterotetramer assembly. Various nerve cells, such as neurons, astrocytes, oligodendrocytes, and microglia, express IDH3β [116]. Reduced ATP biosynthesis results from decreased glucose metabolism, which impairs the generation of an action potential and reduces the capacity of neurons to maintain ionic gradients. Disorders in glucose metabolism play crucial roles in the development of AD because they cause mitochondrial malfunction and neuronal death when this ionic gradient is disrupted, which results in an extracellular influx of Ca^2+^.

Recent research has revealed that IDH3β levels are significantly reduced in the brains of AD animal models and patients with AD [117]. Intracellular lactate accumulates due to TCA cycle impairment, oxidative phosphorylation coupling dysfunction, and reduced IDH3β protein levels. The buildup of lactate increases the expression of Kla and PAX6. Elevated PAX6 further suppresses IDH3β expression, creating a detrimental positive feedback loop [68]. This feedback loop contributes to tau protein hyperphosphorylation, Aβ accumulation, and synaptic damage. By upregulating IDH3β and downregulating PAX6, cellular energy metabolism can be improved, and histone Kla levels can be reduced, thereby alleviating AD-like pathology and cognitive deficits [76]. Overall, the accelerated accumulation of lactate enhances the IDH3β-lactate-PAX6-IDH3β positive feedback loop, suggesting that IDH3β is a novel molecular target for the treatment of AD.

#### 4.2.4. Exercise

Lactate is the primary byproduct of exercise. It is carried by the blood to different bodily tissues. MCTs carry it to various parts of the brain, including the hippocampus, across the blood–brain barrier [118]. Exercise is suggested as a nonpharmacological approach for the prevention and treatment of neurodegenerative illnesses because it is linked to neuronal protection and anti-aging [119]. By altering the chromatin structure, the lactate generated after exercise can trigger Kla and control gene transcription and expression [120].

Aluminum is a neurotoxin linked to the onset of AD, whereas D-galactose (D-gal) is an aging agent. D-gal/aluminum chloride (D-gal/AlCl_3_) can cause symptoms similar to AD, such as amyloid overexpression, oxidative damage, microglial activation, neuroinflammation, and cognitive and memory impairment [121]. The development of AD is significantly influenced by microglia. Restoring phenotypic microglia can slow the course of AD and enhance neuroprotective activity [122]. Exercise can slow the progression of neurodegenerative diseases; some studies have suggested a connection between exercise training and improved cognitive function [77]. Lactate is a novel endogenous metabolite that links exercise to enhanced cognitive function. The study found increased lactate levels following exercise in the mouse brain and plasma. Exercise training enhanced cognitive performance in aged and D-gal/AlCl_3_-treated mice by lowering neuroinflammation. Exercise may help avoid neuroinflammation in the CNS by controlling the activation status of microglia. By acting as an “accelerator” of the “lactic acid timer” in microglia, the increase in lactate levels in the brain following exercise encourages the transformation of microglia to the repair phenotype through histone Kla, thereby inducing the M2 repair phenotype transformation of AD microglia and lowering neuroinflammation [77].

### 4.3. Acute Ischemic Stroke (AIS)

AIS is the leading cause of mortality and disability among adults and a commonly occurring condition. AIS has a major negative impact on patients’ quality of life and health [123]. The standard treatment for AIS is an early resumption of blood reperfusion and early endovascular treatment to save lives and reduce complications [124]. Cerebral ischemia and reperfusion injury is a process in which blood vessels return to normal blood flow, resulting in increased or new damage to cells or tissues in this part of the blood supply area. Due to the substantial depletion of intracellular glycogen reserves during ischemia, the rapid supply of glucose following reperfusion may exceed the cell’s oxidative metabolic capacity, leading to excessive activation of the glycolytic pathway and increased lactate production [125]. Energy for brain metabolism primarily comes from glucose OXPHOS. Acidosis and brain edema may result from the buildup of lactate, which can lead to the loss of bicarbonate anions and accumulation of lactate anions [126]. Recently, studies on the mechanism of cerebral ischemia and reperfusion injury have improved, mainly involving glutamate excitotoxicity, cellular energy failure, oxidative stress, calcium overload, apoptosis, acidosis, inflammatory reaction, excessive production of free radicals, oxidative stress, mitochondrial dysfunction, and blood–brain barrier destruction [127].

In this regard, the newly discovered Kla process sheds light on the pathophysiology associated with lactate metabolism. Increased lactate production during cerebral ischemia indicates a malfunction in energy metabolism, and severe acidosis can result from the buildup of lactate brought on by decreased cerebral blood flow. Lactate can be utilized as a fuel to promote brain function, according to other research [128]. In the mouse model of permanent middle cerebral artery occlusion (MCAO), intravenous injections of sodium lactate for 24 h following ischemia or 3 h following ischemia have a protective effect and improve the prognosis of the nervous system [129]. However, lactate may only have positive effects during certain time frames that are tightly linked to metabolism at certain points after initiation. The role of histone Kla in preserving immunological homeostasis and tissue repair in ischemia and reperfusion injury has drawn interest [130]. Exploring the relationship between lactate and lactylation, as well as the role of lactylation in the pathological process of AIS, may provide a theoretical basis for the development of new therapeutic strategies.

#### 4.3.1. HDAC6

Achieving self-repair of neural tissue following cerebral ischemia and reperfusion injury remains a hot topic in contemporary research. Neuroinflammatory reactions can aggravate cell damage and play a key role in AIS. According to recent research, lactate can operate as an energy substrate for injured neurons for a predetermined period of time following the onset of AIS [62]. Different immune cells in the peripheral blood enter the ischemic area of the brain as a result of damage to the blood–brain barrier. Furthermore, immune cells, including macrophages, gather locally at the lesion site, which leads to immune cells infiltrating the injured brain tissue and worsening brain damage. Histone Kla affects the polarization of macrophages from proinflammatory M1 to reparative M2 [131]. The inflammatory response is an important factor in brain ischemia–reperfusion damage. Lactate production and histone Kla levels increase during reperfusion. In the subacute or late stage, accumulated lactate molecules regulate macrophages from an inflammatory phenotype to a repair phenotype by histone Kla to maintain cell homeostasis, reduce cerebral ischemia–reperfusion injury, and improve the prognosis of AIS [132].

HDAC6 is a unique member of the HDAC family that targets cytoplasmic nonhistone substrates, such as α-tubulin, contact proteins, and heat shock protein 90. Studies have shown that HDAC6 can cause neuronal damage in nervous system diseases, including AIS [133]. HDAC6 is associated with the deacetylation of the cytoskeletal protein α-tubulin, thereby disrupting the stability of microtubules and leading to the dysfunction of vesicle transport in neurons [134]. It causes neuron synaptic dysfunction by obstructing mitochondrial transport at certain sites [135]. HDAC6 can control the degree of oxidative stress and inflammatory state of the cells by controlling protein deacetylation or ubiquitination [136]. Lactate enhances Kla, thereby exerting a neuroprotective effect in cerebral ischemia and reperfusion injury. Neuronal damage is linked to the Kla of HDAC6 in cerebral ischemia and reperfusion injury. By binding to the immunoglobulin heavy chain binding protein, HDAC6 Kla alters calcium homeostasis, further controlling the dendritic architecture of neurons following AIS and guaranteeing the survival of more neurons. Ischemic neuron damage worsened if the Kla level of HDAC6 in the neurons is downregulated [78].

In conclusion, evidence from mouse models and patients with AIS indicates the significance of lactate in neurological prognosis. A fresh viewpoint on lactate processes and fresh concepts for interpreting AIS injury pathways are offered by HDAC6Kla, which targets immunoglobulin heavy chain binding protein-related calcium homeostasis. HDAC6Kla will be considered an important therapeutic target for AIS nerve damage.

#### 4.3.2. Lymphocyte Cytoplasmic Protein 1 (LCP1)

Following AIS, cerebral blood circulation is disrupted, and local tissues experience hypoxia and ischemia damage, which frequently results in neuronal loss and eventually, neurological deficiency symptoms, such as aphasia and paralysis [137]. Recent studies have indicated that after AIS, the lactylation level of LCP1 is significantly enhanced. This exacerbates neuroinflammation and brain injury by modulating the metabolism and function of immune cells, further promoting the development of AIS [79]. Inhibiting glycolysis can reduce the lactylation level of LCP1 and degrade LCP1, thereby alleviating the progression of AIS [138]. The actin-binding protein LCP1 primarily controls cell migration by interacting with actin. It is one of the primary proteins that bind to the cytoskeleton and directly regulate cell mobility [139]. LCP1 plays a role in T cell activation, immunological synapse development, host defense, etc. Glycolysis is minimal in neurons under normal conditions, but when AIS occurs, aerobic respiration is suppressed, and glycolysis is increased in the neurons to preserve the energy supply [140]. Nevertheless, neurons maintain a high rate of glycolysis during the reperfusion phase, when blood oxygen is restored. This phenomenon is called the Warburg effect. Even with adequate oxygen, neurons continue to prefer glycolysis as an energy source, exacerbating neuron death [141].

Following AIS, an increase in vascular permeability, aberrant lactate metabolism, and significant blood lactate accumulation has been observed. Rats treated with MCAO and PC12 cells activated with oxygen glucose deprivation/reoxidation were used to create in vitro and in vivo models of AIS. MCAO rats had higher levels of Kla and total lactylation of LCP1. LCP1 deletion decreased the rate of apoptosis and increased the viability of PC12 cells induced by oxygen glucose deprivation/reoxidation. Additionally, after receiving the glycolysis inhibitor 2-DG, the Kla levels in LCP1 were markedly decreased. In conclusion, inhibiting glycolysis lowers the Kla level of LCP1, which causes LCP1 to degrade and eventually slows the onset of AIS [79]. These findings imply that LCP1 advances AIS through lactate-mediated Kla changes, offering fresh approaches to AIS treatment in the future.

#### 4.3.3. Low-Density Lipoprotein Receptor-Related Protein-1 (LRP1)

The endocytotic receptor, known as low-density LRP1, controls various cellular processes, such as cell survival, differentiation, and proliferation. Various ligands, such as Aβ, apolipoprotein E, and activated α2 macroglobulin, are transported and degraded by lysosomes through the action of LRP1 [142]. LRP1 has a significant impact on neuronal function and early development by influencing synaptogenesis and neuronal activity. By lowering lactate production and ADP-ribosylation factor 1 (ARF1) Kla, astrocyte LRP1 facilitates mitochondrial transport from astrocytes to neurons and ameliorates cerebral ischemia and reperfusion injury. ARF1 is a cytoplasmic protein that promotes vesicle trafficking. LRP1 increases the release and transport of healthy mitochondria to neurons and decreases ischemia and reperfusion injury in a mouse AIS model by inhibiting glycolysis, lactate generation, and ARF1 Kla in astrocytes [80]. In summary, astrocyte LRP1 transfers mitochondria to the neurons and alleviates AIS by inhibiting ARF1 Kla. Future studies can better help understand the neuroprotective impact of LRP1 and investigate its relevance and therapeutic potential in the CNS by monitoring mitochondrial mobility between cells.

### 4.4. Schizophrenia (SCZ)

SCZ is a crippling neuropsychiatric condition that is impacted by both environmental and hereditary causes. SCZ presents with various symptoms as follows: (1) cognitive disorders that impact learning, attention, executive function, and memory, particularly working memory; (2) negative symptoms, such as emotional passivation, social disengagement, and deficiencies in motivation and reward processing; and (3) positive symptoms, such as delusions, hallucinations, paranoia, thinking disorders, and psychomotor restlessness [143]. Cognitive dysfunction is a core feature of >80% of the patients with SCZ [144]. Environmental variables have a major impact on how CNS develops and functions as well as on how mental disease manifests [145]. Both genetic and environmental variables can contribute to SCZ, and epigenetic changes, particularly those involving histones, may play a role in its development [146]. Epigenetic modifications alter chromatin shape, which controls gene transcription rates, but they do not alter DNA sequence [147]. Changes in glycolysis and metabolite levels may be linked to the death of hippocampal neurons in SCZ. Glycolysis-related enzymes are highly expressed in the hippocampal tissues of patients with SCZ, according to mass spectrometry analysis, and the amount of glycolysis-related products varies in patients’ brains [148]. Lactate—a significant byproduct of glycolysis—is elevated in SCZ [149]. Increases in lactate and glycolysis in hippocampal neurons may result in alterations in neuronal function and cell death because lactate encourages neuron activation [150]. Therefore, lactate may play a role in SCZ pathophysiology.

A highly conserved nonhistone chromosomal-binding protein, high mobility group box 1 (HMGB1), is assumed to be a nuclear DNA-binding protein involved in gene transcription and nucleosome stability. An increase in HMGB1 is an indicator of SCZ [151] and may impact disease pathophysiology by triggering downstream pathways that control inflammatory responses. The malfunctioning of hippocampal neurons is linked to an increase in HMGB1 [152]. Abnormal hippocampal function is one of the main causes of symptoms in patients with SCZ. SCZ progression can be halted by blocking apoptosis in hippocampal neurons [153]. MK801 induces increased glycolysis and Kla levels, most notably in mouse oligodendrocytes, in SCZ mouse models [154]. In the MK801-induced SCZ paradigm, HMGB1 was upregulated in primary hippocampal neurons, and increased HMGB1 may have caused apoptosis in hippocampal neurons [155]. Furthermore, lactate accumulation and elevated levels of histone H3K9la and H3K18la lead to increased HMGB1 levels in the hippocampus and behavioral changes in SCZ model mice. But therapeutic administration of 2-DG, a glycolysis inhibitor, can lessen behavioral alterations in the SCZ mouse model by lowering lactate buildup and histone H3K9la levels [81]. This finding indicates that H3K9la may be a key factor of glycolysis-induced lactylation modification in SCZ.

In conclusion, a thorough understanding of the pathophysiology of SCZ will improve its therapy, investigate the involvement of Kla in the development of SCZ, and possibly identify targets for early clinical diagnosis and treatment.

## 5. Future Directions

This study summarizes the production and utilization of lactate in the nervous system and its role and mechanism in the CNS. The impact of lactate on brain function is complex and diverse. New theories on the role of lactate in the physiology and pathology of CNS diseases have been offered by the discovery of Kla, which uses lactate as a substrate. Studies have investigated its roles and regulatory mechanisms in the incidence and progression of various diseases. Although there have been some studies on the regulatory mechanisms of Kla, lactylation sites and their effects, and interactions with other PTMs, overall, research on the entire protein Kla is in its infancy, and more basic experiments and clinical research will be needed in the future. The findings will provide more accurate information and fresh ideas for treating CNS diseases.

## Figures and Tables

**Table 1:** The role of lactate and lactylation in central nervous system diseases.

Diseases	Target	Mechanisms	References
Traumatic brain injury	GPR81	Lactate activates GPR81, improving synaptic function and promoting neuronal protection.	[73,74]
Alzheimer’s disease	H4K12la	glycolysis/H4K12la/PKM2 feedback loop exacerbates microglial dysfunction.	[54]
	H3K18la	The H3K18la/NFκB axis promotes AD pathology by regulating SASP components IL-6 and IL-8.	[75]
	IDH3β	Lactate enhances the IDH3β-lactate-PAX6 loop, worsening AD pathology and cognition.	[76]
	exercise	Increased brain lactate after exercise promotes microglial transformation to the M2 repair phenotype via histone Kla, reducing neuroinflammation in AD.	[77]
Acute ischemic stroke	HDAC6	By binding to the immunoglobulin heavy chain binding protein, HDAC6 Kla alters calcium homeostasis, controlling dendritic architecture and promoting neuronal survival after AIS.	[78]
	LCP1	LCP1 modulates immune cells to worsen neuroinflammation and drive AIS progression.	[79]
	LRP1	LRP1 reduces lactate and ARF1 Kla, boosts mitochondrial transport to neurons, and improves AIS.	[80]
Schizophrenia	H3K9la	Lactate accumulation and elevated H3K9la/H3K18la levels increase hippocampal HMGB1 and induce behavioral changes in SCZ mice, yet 2-DG treatment reduces these alterations by lowering H3K9la levels.	[81]

## Abbreviations

The following abbreviations are used in this manuscript:

PK/PYK	Pyruvate kinase
OXPHOS	Phosphorylation
LDH	Lactate dehydrogenase
CNS	Central nervous system
MCT	Monocarboxylate transporter
GPCR	G protein-coupled receptor
Glut1	Glucose transporter 1
PTMs	Post-translational modifications
Kla	Lysine lactylation
HDAC	Histone deacetylase
PKM2	M2 isoform of PK
TBI	Traumatic brain injury
BDNF	Brain-derived neurotrophic factor
AD	Alzheimer’s disease
Aβ	Amyloid beta
H4K12la	H4k12 lactylation
H3K18la	H3k18 lactylation
SASP	Senescence-associated secretory phenotype
TCA	Tricarboxylic acid
IDH3	Isocitrate dehydrogenase 3
D-gal	D-galactose
AIS	Acute ischemic stroke
LCP1	Lymphocyte cytoplasmic protein 1
MCAO	Middle cerebral artery occlusion
LRP1	Lipoprotein receptor-related protein-1
ARF1	ADP-ribosylation factor 1
SCZ	Schizophrenia
HMGB1	High mobility group box 1
